# Biofouling Growth in Cold Estuarine Waters and Evaluation of Some Chitosan and Copper Anti-Fouling Paints

**DOI:** 10.3390/ijms10073209

**Published:** 2009-07-14

**Authors:** Émilien Pelletier, Claudie Bonnet, Karine Lemarchand

**Affiliations:** Institut des Sciences de la Mer de Rimouski, Université du Québec à Rimouski, 310 Allée des Ursulines, C.P.3300. Rimouski, QC, G5L 3A1, Canada; E-Mails: claudie.bonnet@nrc-cnrc.gc.ca (C.B.); Karine_lemarchand@uqar.qc.ca (K.L.)

**Keywords:** marine biofouling, copper antifouling paint, chitosan, static field testing, microalgae

## Abstract

Ecological concerns about antifouling paints containing non-green tin and copper compounds have highlighted the need for environmentally friendly alternatives. We report here a field test conducted in estuarine waters over two months designed to evaluate the efficiency of a number of active natural and man-made chemical ingredients added into a silicon-polyurethane marine paint. Early steps of biofouling in cold seawater of the St. Lawrence Estuary (Canada) were observed. Analyses, including dry biomass, flow cytometry and spectrofluorimetry, demonstrated a short-term antibacterial action of chitosan-based paints although no significant anti-algal action was observed. Cuprous oxide paints were efficient against bacteria and algae invasion in the first two weeks, especially those with added organic biocides such as isothiazolone and copper pyrithione. However, the overall dry biomass and chlorophyll *a* content were similar for all chitosan-and copper-based paints after 63 days. Microscopic observations revealed variation in the highly diverse benthic diatom population including species *Navicula*, *Melosira*, *Cocconeis*, *Nitshzcia*, *Fragilaria* and *Amphora*. Results suggest no real long-term efficiency for tested antifouling paints and highlight a particular need for green antifouling ingredients that are active under northern estuarine conditions.

## 1. Introduction

Being a source of environmental and economical problems, the invasive biofouling process creates adverse effects on all immersed structures limiting their utilization and accelerating their corrosion. Of all the solutions proposed to prevent biofouling, tributyltin (TBT) self-polishing copolymer paints were by far the most successful solution and were used on more than 70% of the world commercial fleet in mid-1990s [1]. Unfortunately, TBT was found to be the most toxic substance ever introduced in the marine environment, with harmful effects to non-target organisms [2]. Tin-free alternative antifouling chemical agents for use in the aquatic environment have been a topic of increasing concern over the past years as stable degradation products could cause environmental problems by their increasing concentrations [1,4]. A number of natural products were investigated for their antifouling potential. Compounds isolated from marine organisms such as bacteria, algae, cnidaria, bryozoa, chordate and other sessile benthic invertebrates were tested against biofouling [4,5,6]. A recent example is given by Tsoukatou *et al*. [7], where sponge metabolites and their synthetic derivatives were tested in laboratory against fouling organisms.

Among organic polymers highly abundant in marine crustaceans, chitosan, obtained from the partial deacetylation of chitin, possesses antimicrobial properties against a number of organisms, including bacteria, fungi and algae [8,9]. Chitosan could be part of a green chemistry approach to fight biofouling as it comes mainly from seafood by-products and its preparation requires a limited number of steps. Although a large number of laboratory tests and assays have been developed to assess the activity of organic and mineral compounds against fouling organisms [7,10,11] few practical field tests have been described [12], particularly in cold waters where the fouling process is slow.

The first aim of this work was to test the efficiency of finely ground (60 mesh) chitosan incorporated as an antifouling additive into a polyurethane marine paint lacking any other biocide. The second objective of this work was to develop a practical *in situ* test for estuarine waters to describe early steps of biofouling on painted metal plates and quantify its progress over a two-month static exposure. Never assessed before, the effectiveness of chitosan granular polymer as an additive to marine paint is reported and compared to three commercially available antifouling marine paints.

## 2. Results and Discussion

The early microbial biofouling community has never been described in the St. Lawrence Estuary (Eastern Canada) where cold waters persist year-round. The temporal sequence of bacterial-algal communities in the slime film developing on a surface immersed in the Atlantic seawater was detailed in early 1950s in a book published by the Woods Hole Oceanographic Institute where bacterial concentrations were reported to reach 10^6^ organisms cm^−2^ within two days [21]. These values are far higher than the counts (10^4^–10^5^ bacteria cm^−2^) obtained in the present study after 14 days. Meteorological conditions prevailing in this northern estuary, and especially its low water temperature, are probably responsible for the slow bacterial colonization. Even under these low growth rate conditions, a thin sticky biological layer was observed on every panel in the first three weeks.

### 2.1. Bacterial Counts

Paint compositions and abbreviations are given in Table 1. *CuO, Oma* and *SN* are copper-based paints, whereas *C1* and *C2* are chitosan-based paints.

**Figure 1.**
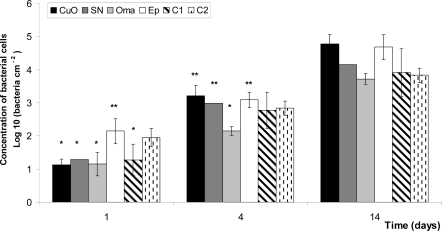
Concentration of bacteria cm^−2^ after 1, 4 and 14 days. *CuO, Oma* and *SN* are copper-based paints, whereas *C1* and *C2* are chitosan-based paints and *Ep* is the paint without additive. Standard deviation is shown with error bars. Columns with one asterisk (*) present values not different to each other or to columns without asterisks, but are significantly different to values from columns with two asterisks (**). Columns without asterisk are not significantly different to each other for the same day.

The relatively low bacterial count for all plates is probably due to the low temperature, as nutrients were abundant and typical of estuarine conditions. The bacterial abundance per cm^2^ increased by about four orders of magnitude within the first 14 days after immersion (Figure 1).

After the first 24 h of exposure, the free-biocide paint, *Ep*, already presented a significantly higher concentration of surface bacteria than the anti-fouling paints *C1, CuO, Oma* and *SN*. Data show a weaker bacterial attachment on the chitosan paint *C1* (5% w v^−1^) compared to commercial antifouling paints. After 4 days, *Oma* presented a significantly lower number of bacterial cells than *CuO, Ep* and *SN*, but did not present significant differences with *C1* or *C2*, suggesting an antimicrobial action of both chitosan formulated paints. After 14 days, the tendency of *Oma* and *C2* (20% w v^−1^) panels to be less colonised by bacteria is still visible, but was not significantly different from others.

### 2.2. Algae Counts and Chlorophyll A Content

**Figure 2.**
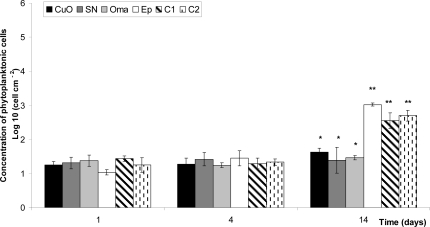
Concentration of phytoplanktonic cells cm^−2^ after 1, 4 and 14 days. Standard deviation is shown with error bars. Meaning of asterisks is as given in Figure 1.

Concentrations of phytoplankton cells settled on painted panels in the first two weeks were relatively low, between 60 to 130 cells cm^−1^ (Figure 2). Significant differences in the number of attached cells cm^−2^ between panels only appeared after 14 days. Panels *Ep, C1* and *C2* presented cell concentrations much higher than copper formulated antifouling paints. Copper paints with additives (*SN* and *Oma)* seem less efficient than the free-additive copper paint *CuO* (Figure 2).

**Figure 3.**
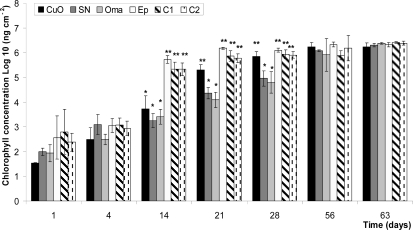
Chlorophyll *a* in ng cm^−2^ after 1, 4, 14, 21, 28, 56 and 63 immersion days. Standard deviation is shown with the error bars. Meaning of asterisks is as given in Figure 1.

Chlorophyll *a* (Chl a) concentrations on the surface of tested panels increased by four orders of magnitude over the exposure period of 63 days (Figure 3). Chl *a* results after 14 days also reveal the lack of anti-algal efficiency for polyurethane-silicone based paints (*Ep, C1* and *C2*), as the number of cells cm^−2^ is over 10 times higher than copper paints. Although a comparable efficiency of *CuO, SN*, and *Oma* is observed on day 14, important changes are visible in the following two weeks as paints with additives (*SN* and *Oma*) are significantly more efficient at day 21 and day 28. However, the final sampling showed concentrations reaching 10^6^ ng cm^−2^ for all panels without differences between paints.

### 2.3. Total Dry Biomass

**Figure 4.**
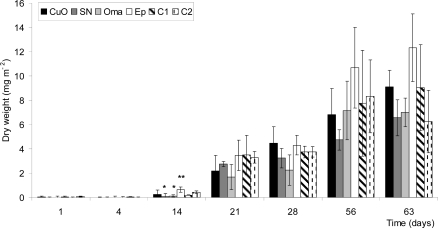
Dry weight biomass in mg m^−2^ after 1, 4, 14, 21, 28, 56 and 63 days. Standard deviation is shown with the error bars. Meaning of asterisks is as given in Figure 1.

An increase of the dry fouled biomass in time was undeniable (Figure 4). A significant difference was only detected after 14 days between *Ep* and the cuprous oxide paints with added biocides, *SN* and *Oma.* High heterogeneity in biofouling on test panels and a limited number of replicates precluded a determination of significant differences in the other sampling periods. *Ep* paint seemed to exhibit the highest biofouling dry weight after 63 days (P=0.065). Overall, the total biomass weighted was similar for all paints even after 63 days and averaged 8.38 mg m^−2^.

The results of above methods to estimate biofouling growth were compared using two-way ANOVA and differences (p≤0.001) were observed in time between some paints (Table 2). All four parameters examined can easily discriminate changes with time and between paints but the combining effects of time and paints are best described by chlorophyll a and phytoplankton count.

### 2.4. Visual and Microscopic Observations

Painted panels presented little evidence of biofouling after three weeks of immersion, although a thin slime layer was present on the surface. A few macroalgal structures became visible in the fourth week, brown and sticky on *CuO* panels, hairy dark green on *C1, C2* and *Ep.*

**Figure 5.**
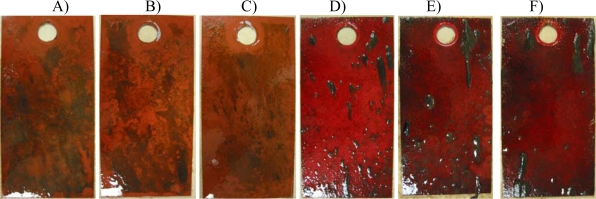
Panels as they appeared after 56 days of immersion. A) *CuO,* B) *SN*, C) *Oma*, D) *Ep,* E) *C1*, F) *C2.*

Those macro-structures appeared to be formed by aggregated colonies of benthic diatoms of hard substrate and hypothesised to be *Amphora* spp. on *CuO* and suggested to be formed of pennales diatoms (*Navicula* spp., *Nitszchia* spp. or *Berkeleya* spp.) on *Ep* based paints (not shown). After 56 days, all panels were covered with a layer of macoralgae (Figure 5). A difference in colonising algae between cuprous oxide based paints (Figures 5 A, B and C), forming a brownish layer, and polyurethane-silicone based paints (Figures 5 D, E and F), with hairy dark green aggregates, was quite evident. Macro-invertebrates such as gastropods, tunicates, and polycheates were not observed on panels even after two months of immersion.

**Figure 6.**
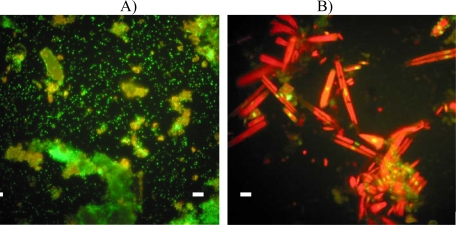
Epifluorescence microscopy 60X after 21 immersion days. A) *SN*; B) *C2*. Bacteria appear as green dots and microalgae as red sticks. Debris appears in greenish-yellow. White bar: 10 μm.

Epifluorescence microscopy performed on the organic material harvested on painted panels revealed the formation of a filamentous biofilm after 14 days of immersion on epoxy-paints, *C1, C2* and *Ep* and some tiny patches on *CuO* whereas *Oma* and *SN* panels were not yet colonised by microalgae in accordance with their very low chlorophyll *a* concentrations. After three weeks, *C1, C2* and *Ep* panels were completely covered with an algal film, composed of various species of benthic diatoms (Figure 6B). Copper painted panels showed well developed bacterial clusters although algae were still not visible (Figure 6). After 63 days, all panels were intensely colonized by algae and bacteria with no visual differences between antifouling paints.

**Figure 7.**
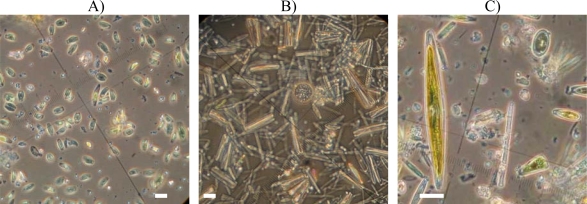
Inverted microscopy observations, 40X, after 63 days. Nitric and sulphuric acid treatment. A) *Oma,* B) *C2*. Without acid treatment: C) *Navicula directa*, observed on *Ep.* White bar: 10 μm.

Observations using inverted microscopy on samples immersed over 63 days confirmed the presence of two different communities involved in the biofouling process of either cuprous oxide based paints (*CuO, SN* and *Oma*) or *Easypoxy*^™^ based paints (*Ep, C1* and *C2*). Microalgae *Amphora* spp. Were dominated on cuprous oxide paints (Figure 7A). On *Ep* paints a high diversity of microalgae are visible (Figure 7B). Diatoms such as *Navicula* spp., *Melosira* spp., *Cocconeis* spp., *Nitzshcia* spp., *Fragilaria* spp., and *Amphora* spp. were observed and identified [20]. Specific identification of *Navicula directa* and *Melosira nummuloides* was even possible (Figure 7C).

### 2.5. Statistical Treatment

**Figure 8.**
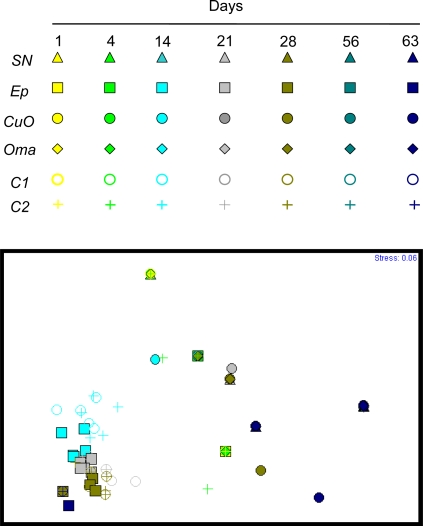
Structure of the whole assemblage, nMDS ordination on presence-absence data, using Bray-Curtis similarity matrix. Bacteria, bacterial clusters and variation of microalgae morphology observed. Stress ≤ 0.2 demonstrates a good graphical representation of the differences and similarities between paints.

Based on photographic records obtained from epifluorescence microscopy, a non-metric multivariate analysis based on the presence and the absence of different microorganisms was performed. Figure 8 shows the differences and similarities of colonisation between the paints in time.

Each paint type is represented by a specific symbol and each sampling day by a different color. Paint types similarly populated by microorganisms have their respective symbols closer to each other. Although some symbols are hidden by others, it is possible to note that *Ep, C1* and *C2* symbols are clustering in the lower left corner. Those painted panels appear to be similarly colonised by organisms in time. The symbols of *CuO, Oma* and *SN* paints are more spread but still relatively closer to each other. Cuprous oxide based paints are similarly populated but are not related to *Easypoxy*^™^ based paints. Symbols of *C2* paint are sometimes superimposed on *Oma* symbols, but at different times showing a different biofouling progression on the surface of the paints. The stress indication of 0.06, far under the 0.2 usually admitted, reveals an excellent graphical representation of the differences and similarities between the paints in time.

### 2.6. Tools to Assess Field Biofouling

Chlorophyll *a* content is a good and simple tool to estimate the evolution of algal biomass with time, but does not allow the differentiation between species involved in the colonisation process of painted panels. Visual observations showed differences of alga genus colonising cuprous oxide based paints and *Easypoxy*^™^ based paints. Those variations in composition of the microalgal communities were confirmed by epifluorescence and inverted microscopy. A higher number of species were visible on *Ep* paints, diatoms such as *Navicula* spp., *Cocconeis* spp., *Nitzschia* spp., *Melosira* sp., *Fragilaria* spp., *Licmophora* spp. and some species of *Amphora* spp. were observed and identified. The frequent occurrence on test surfaces of genus *Licmophora* spp., *Navicula* spp. and *Nitzschia* spp., was previously reported [21], but types and numbers of native fouling organisms differ within regions, especially in regions where marked seasonal variations in temperature occur [21]. The diatom population on cuprous oxide paints was mainly composed of *Amphora* spp. This result was expected and confirms the resistance of this genus of diatoms to copper toxicity [22]. Surprisingly, *Amphora* also appears to be resistant to the organic biocides *Oma* and *SN*.

Dry weight analyses include living and non-living organisms and give a good estimation of the total biomass of biofouling with time. Paints did not present significantly different values of total biomass on their surface throughout the experiment, except on day 14. However, a tendency of *Ep* paint to present higher values of dry weight is noted. Furthermore, chitosan paint *C2*, as well as *SN* and *Oma* presented the lowest biomass values after 63 days of immersion. Values obtained were really small and the high heterogeneity between panels and on the same panels, especially when macroalgae started to be abundant, explains the great variability between replicates. In spite of these methodological restrictions in the experimental setup, it can be concluded that painted panels immerged nine weeks in northern cold seawater showed low and similar total dry biomass and algal concentration with or without toxic compounds.

### 2.7. Efficiency of Antifouling Components

The control of the early stage of the biofilm, such as the settling of bacteria, fungi and benthic diatoms, should prevent the settlement of subsequent biofoulers such as macro-algae and larvae [5]. Several studies reported the incorporation of natural products in antifouling paint formulations [4–7,23]. However, no attempts have been yet reported to evaluate the potential of solid phase chitosan polymers as antimicrobial additives to a polyurethane-silicon marine paint.

The antimicrobial activity of chitosan has never been directly evaluated in the marine environment as a paint additive so the best effective deacylation degree, molecular weight and concentration for an antibacterial action were unknown. Chitosan presenting a degree of deacylation of 100% was chosen for our experiment as it was previously demonstrated that the deacylation level is correlated to antimicrobial properties [24]. As revealed in numerous studies [8,25], the chitosan molecular weight has an influence on its solubility and antimicrobial activity. A 450 KDa easily produced polymer was chosen for the evaluation of the antifouling potential of chitosan. The solubility of chitosan in silicon-polyurethane paint is unknown and field tests are essential to estimate the real behaviour of the coating once immersed as it could be influenced by various environmental changes. Chitosan *C1* (5% concentrated) presented an interesting antibacterial action after 24 h, but *C2* (20% concentrated) seems to present a more promising antimicrobial action. With concentrations below 2% w v^−1^ chitosan presents antibacterial activity against some genera of microorganisms involved in the biofouling process such as *Pseudomonas, Vibrio* and *Bacillus* [8,24,26]. Our own tests using solid-phase chitosan demonstrated that 5% w v^−1^ presented some antibacterial characteristics, but effects of the 20% w v^−1^ composition seems to last longer. Chitosan polymer has also been reported to control the growth of algae [27], but its action was never directly tested in the marine environment as an anti-fouling additive. Within the present experiment the anti-algal activity of the polymer of chitosan was not demonstrated and painted panels presented an algal growth closely similar to panels painted with control *Ep*. No attempt was made to optimize the concentration of chitosan in the present work.

A much better antibacterial activity of the additive *Omadine*^™^ over all other paints was demonstrated. Results are in agreement with the technical bulletin published by Arch Chemicals, Inc. [28], where Copper *Omadine*^™^ is reported to exhibit pronounced growth inhibiting activity against a broad spectrum of both Gram negative and Gram positive bacteria, as well as fungi and yeast and to outperform all paints containing only cuprous oxide. All cuprous oxide based paints demonstrated an anti-algal action, with *SN* and *Oma* additives being more efficient than any other paint for the first four weeks. Molino *et al*. [29] reported recently the role played by bacterial community during the early stages of colonisation on immerged surfaces and compared a number of antifouling paints in field tests held in temperate and tropical waters. They observed a rapid modification of the coating surfaces (as fast as four days) and a correlation with both location and season. Diatoms dominating in the microfouling biofilm are known to be highly resistant to copper antifouling paints [30], but not to organic biocides added to inhibit the growth of resistant algae such as *Enteromorpha* spp., *Ectocarpus* spp., and *Achnanthes* spp. [31]. Moreover, *Sea-Nine® 211* is claimed to have a broad spectrum activity against diatoms, algae, barnacles, tubeworms, hydroids, bryozoans and tunicates [32]. However, all paints, with or without additives, presented on their surfaces similar concentrations of chlorophyll *a* pigment after two months, suggesting the absence of a real long-term anti-algal action of organic biocides paints in cold estuarine seawaters. Results may have been different in a dynamic test where strong currents may have removed a part of the biofilm.

## 3. Experimental Section

### 3.1. Settlement Device

Steel panels of 10 cm x 20 cm with a 2 cm diameter centered hole at 1 cm from the top were used. Abrasive blasting, cleaning and drying of the panels were performed before their pre-treatment with primer coating (Interprotect 2000E® from Interlux, Ltd). Panels were sprayed on both sides with marine paints then vertically and randomly framed in triplicate on ABS (Acrylonitrile Butadiene Styrene plastic) tubes from the exposure racks following a protocol proposed by the American Society for Testing Materials [12]. Each rack, supporting 24 panels, was attached to the floating raft of the Rimouski harbour (48°28’ N 68°30’W) along the St. Lawrence Estuary with a south west sun exposition and immersed at 50 cm below the water level as suggested by Stupak *et al*. [13].

### 3.2. Weather and Seawater Conditions

The water temperature at Rimouski pier fell from 13.7°C to 7.1°C during the experiment period and a solar radiation decrease from 47.5 E m^−2^ day^−1^ to 17.5 E m^−2^ day^−1^ was observed as the experiment was conducted through summer and autumn conditions from August 12^th^ to October 14^th^ 2005. A total of 243 mm of rain were measured during the 27 raining days recorded over the two months of experimentation. Average seawater salinity was 23 ppm and dissolved oxygen stayed near saturation at 9.1 mg O_2_ L^−1^. Nutrient concentrations (phosphates = 0.94 μM; nitrates = 6.6 μM and silicates = 18.3 μM) were typical to estuarine conditions and highly favourable to algal productivity. Suspended particulate matter averaged 3.1 mg L^−1^ during calm conditions, but reached 90 mg L^−1^ under stormy conditions. The mean pH value was 7.96 during dry days and dropped to 6.86 in rainy days. Currents with the Rimouski habour were subjected to tidal pulses and rarely exceeded 3 cm sec^−1^.

### 3.3. Commercial and Chitosan Paints

Chitosan was prepared from waste *Pandalus borealis* shrimp shells by successive deproteinisation, demineralisation and deacylation of chitin. Three commercially available paints, cuprous oxide paint *Bottomkote*® *XXX* (Interlux, Ltd.), liquid *Sea-Nine*®*211* cuprous oxide paint additive (Rohm & Haas Inc., Philadelphia, US.), Copper *Omadine*^™^ powder additive for cuprous oxide paint (Arch Chemicals Inc., Norwalk, US), and two granular chitosan added paints were compared for their antifouling properties (Table 1). *Easypoxy*^™^ polyurethane paint (Pettit Marine Paints Division of Kop-Coat, Inc., Rockaway, US.), a marine paint without antifouling biocide, was used as a referential non-toxic surface. All paints were red to minimise possible color bias on biofouling process.

### 3.4. Sampling Procedure

Seven racks were used over a two-month period and sampled after 1, 4, 14, 21, 28, 56, and 63 days. An exposure rack, containing 24 suspended panels, was removed and inspected at each sampling time. Organic material firmly attached to panels was scrapped off with a sterile razor blade for different analyses [14]. Material collected from a 60 cm^2^ surface was washed with sterile fresh water to remove sea salt and freeze-dried for the determination of dry weight expressed in mg m^−2^. A second 20 cm^2^ surface was scraped off and the organic material removed with sterile standard seawater (SSW: 33 g NaCl L^−1^). The resulting sample was preserved in 2% formalin at −80°C and analysed by flow cytometry for bacterial count, epifluorescence microscopy for bacterial and algal observation, and inverted microscopy for algae identification. Organic material collected from another 20 cm^2^ surface was preserved at −80°C and analysed in spectrofluorimetry for the determination of the chlorophyll *a* content.

### 3.5. Direct Photographic and Microscopic Observations

Digital photographic records were obtained and used to compare biofouling progression. Thawed samples preserved in formalin (500 μL) were stained for 15 min at 20°C in the dark with 0.2‰ SYBR green I (Molecular Probes, Inc.). After staining, bacteria and algae from the biofilm were trapped onto 0.2 μm black polycarbonate membranes (Osmonics, Inc.) and observed with an Olympus BX40 microscope. A 530 nm FITC filter was used for epifluorescence microscopy observation and photographic records. From the same thawed sample, a 100 μL aliquot was observed in inverted microscopy at 40X according to the procedure described by Lund [15] for algae identification and numerical microphotographs were taken. A nitric and sulphuric acid treatment was applied to 1 mL fixed samples for identification of diatoms using inverted microscopy.

### 3.6. Flow Cytometry

Samples preserved at −80°C in 2% formalin were thawed and 1 mL was used for flow cytometry analysis (FACSort, Becton Dickinson™) to reveal the presence of bacteria and algae in the samples. Bacterial population samples were stained with the high-affinity nucleic acid SYBR green I according to procedure previously described [16] and natural fluorescence of the phytoplankton population was detected. An internal standard mixture, consisting of 2 μm diameter beads (Polysciences, Inc.) was added to each sample. Analyses were performed on CellQuest software version 1.0® (Becton Dickinson Immunocytometry Systems). An isolated bacterial population, formed by cells smaller than 2 μm, was detected and measured. Bacterial counts per cm^2^ as a reference of surface colonisation were determined.

### 3.7. Chlorophyll A

Samples preserved at −80°C were thawed and 1 mL was analysed in fluorimetry following Trees *et al*. [17]. Briefly, samples were added in a 90% acetone solution, quickly sonicated and left in dark 24h at 4°C for pigment extraction. Analysis was performed with a Turner Designs Fluorometer, and concentrations expressed in ng of chlorophyll *a* per cm^2^ were calculated using the Strickland and Parsons method [18].

### 3.8. Statistical Tests

Differences in the mean values of biofouling growth in time between each paint were determined using a two-way analysis of variance (ANOVA). Flow cytometry and fluorimetry analyses results were log10 transformed to obtain a normal distribution of frequencies. The location of all racks at the pier raft was randomly distributed and was considered to have no effects on the results. The analysis of variance was followed by a Tukey post-hoc test. Probabilities lower than or equal to 0.05 were considered significant. Using the photographic records obtained from microscopic epifluorescence, multivariate analyses were performed. Differences in the structure of whole assemblages among the six paints were identified by non-metric multi-dimensional scaling (nMDS) ordination on presence-absence data, using the Bray-Curtis similarity measure [19]. The presence or absence of bacteria, bacterial clusters and various morphologies of microalgae were specifically observed (Table 2).

## 4. Conclusions

Methods adapted here to evaluate the biofouling growth in time were useful to understand how colonisation on painted surfaces progresses. Analyses performed on bacterial and phytoplankton populations provided new details on the early stages of biofouling in the St. Lawrence Estuary, rarely investigated before. Microscopic observations provided some specific indications on the algal community composition, information improving our knowledge of the fouling diatoms in the Estuary. Our results question the interest of using antifouling paints and additives organic biocides on ship hulls navigating the St. Lawrence Estuary as the fouling is slow to take place and all paints were equally inefficient after two months. This suggests that expensive and toxic copper paints need not been used on immersed structures in St. Lawrence Estuary as they are useless towards the slow biofouling taking place over summer months. The discovery of a lower bacterial adherence for solid phase chitosan added to a commercial marine paint without any pesticide antifouling compound is encouraging for the development of a green chemistry approach to harmful effects of biofouling. This natural polymer has excellent properties such as non-toxicity, non-allergenicity, biocompatibility and biodegradability [27]. However, in comparison to cuprous oxide based paints with and without additives, chitosan was not efficient against microalgae in the early stage of the biofouling. A much better understanding of chitosan mechanism of action against marine bacteria is needed. The use of chitosan as an antibacterial additive combined to an environmentally safe anti-algal additive justifies further efforts in formulating chitosan-base anti-fouling additives. Optimal concentration and granular size of chitosan should be determined in a further work. The work should also be extended to the settlement of invertebrate larvae already well described in the St. Lawrence Estuary.

## Figures and Tables

**Table 1.** Industrial and chitosan paints used in the field test.

**Industrial Name**	**Identification**
Cuprous oxide paint (*Bottomkote® XXX*, Interlux, Ltd.)	*CuO*
Isothiazolone. *Sea-Nine® 211* (Rohm & Haas Company, Inc., Philadelphia, US), concentrated 5% in copper paint	*SN*
Copper pyrithione, Copper *Omadine*^™^ (Arch Chemicals, Inc., Norwalk, US), concentrated 5% in copper paint	*Oma*
Silicon-polyurethan *Easypoxy*^™^ (Pettit Marine Paints, Inc., Rockaway, US)	*Ep*

Chitosans * added in*Easypoxy*paint*Ep*	

Chitosan 100% of deacylation, 480 KDa, concentrated 5% (w v^−1^)	*C1*
Chitosan 100% of deacylation, 480 KDa, concentrated 20% ( w v^−1^)	*C2*

* All chitosans were pulverized to 60 mesh prior to use.

**Table 2.** Two-way ANOVA comparing the different paints used at the different times sampled for all biological and chemical analyses performed. The data were log 10 transformed for cytometry and fluorimetry.

		**Field assay**			
	
	**Source**	**df**	**MS**	**F-ratio**	**p**
**Bacterial count**	Time 2		32.50	245.20	≤0.001
	Paint 5		0.98	7.37	≤0.001
	Time*Paint 10		0.32	2.40	0.027
	Error 36		0.13		

**Phytoplankton count**	Time 2		4.03	139.20	≤0.001
	Paint 5		0.47	16.21	≤0.001
	Time*Paint 10		0.57	19.68	≤0.001
	Error 36		0.03		

**Chlorophyll a**	Time 6		245.99	420.71	≤0.001
	Paint 5		21.17	36.20	≤0.001
	Time*Paint 30		3.41	5.83	≤0.001
	Error 84		0.59		

**Dry biomass**	Time 6		0.01	99.54	≤0.001
	Paint 5		0.00	4.88	0.001
	Time*Paint 30		0.00	1.46	0.089
	Error 84		0.00		
